# Optimization of the Mechanical Properties and the Cytocompatibility for the PMMA Nanocomposites Reinforced with the Hydroxyapatite Nanofibers and the Magnesium Phosphate Nanosheets

**DOI:** 10.3390/ma14195893

**Published:** 2021-10-08

**Authors:** Mostafa Rezazadeh Shirdar, Mohammad Mahdi Taheri, Mei-Li Qi, Scott Gohery, Nasim Farajpour, Surya Narayanan, Tara Foroozan, Soroosh Sharifi-Asl, Reza Shahbazian-Yassar, Tolou Shokuhfar

**Affiliations:** 1 Department of Bioengineering, University of Illinois at Chicago, Chicago, IL 60607, USA; rsmostafa2@gmail.com (M.R.S.); taheri24@gmail.com (M.M.T.); suryan90@gmail.com (S.N.); 2 School of Transportation Civil Engineering, Shandong Jiaotong University, Jinan, 250357, China; qimeili@sdjtu.edu.cn; 3 Department of Mechanical Engineering, The University of Melbourne, Parkville, VIC 3010, Australia; scott.gohery@unimelb.edu.au; 4 Department of Electrical Engineering, University of Illinois at Chicago, Chicago, IL 60607, USA; nfara006@ucr.edu (N.F.); sshari25@uic.edu (S.S.-A.); 5 Department of Mechanical and Industrial Engineering, University of Illinois at Chicago, Chicago, IL 60607, USA; tforoo2@uic.edu

**Keywords:** PMMA bone cement, response surface methodology, compressive strength, cell viability

## Abstract

Commercial poly methyl methacrylate (PMMA)-based cement is currently used in the field of orthopedics. However, it suffers from lack of bioactivity, mechanical weakness, and monomer toxicity. In this study, a PMMA-based cement nanocomposite reinforced with hydroxyapatite (HA) nanofibers and two-dimensional (2D) magnesium phosphate MgP nanosheets was synthesized and optimized in terms of mechanical property and cytocompatibility. The HA nanofibers and the MgP nanosheets were synthesized using a hydrothermal homogeneous precipitation method and tuning the crystallization of the sodium-magnesium-phosphate ternary system, respectively. Compressive strength and MTT assay tests were conducted to evaluate the mechanical property and the cytocompatibility of the PMMA-HA-MgP nanocomposites prepared at different ratios of HA and MgP. To optimize the developed nanocomposites, the standard response surface methodology (RSM) design known as the central composite design (CCD) was employed. Two regression models generated by CCD were analyzed and compared with the experimental results, and good agreement was observed. Statistical analysis revealed the significance of both factors, namely, the HA nanofibers and the MgP nanosheets, in improving the compressive strength and cell viability of the PMMA-MgP-HA nanocomposite. Finally, it was demonstrated that the HA nanofibers of 7.5% wt and the MgP nanosheets of 6.12% wt result in the PMMA-HA-MgP nanocomposite with the optimum compressive strength and cell viability.

## 1. Introduction

Over the past fifty years, PMMA has been utilized in odontology and orthopedic applications as the main material for fixation of prosthesis to transfer forces from bone to prosthesis [1,2]. Although commercial PMMA is commonly used as a grounding material in joint replacement surgeries, acrylic bone cements, and anchoring of hip prostheses [3,4], several disadvantages such as lack of bioactivity, mechanical weakness, and monomer toxicity limit its applications [5,6,7,8]. Lack of bioactivity in the PMMA bone cement makes it a bioinert material that prevents chemical bonding with the bone tissue at the implant site and hence does not adhere to the bone [9,10,11]. Weak combination between the PMMA cement and the host bone results in osteolysis and further aseptic loosening or even dislodgement of the bone cement implants [12]. In addition, there are reports on cytotoxicity of PMMA and its components in vitro [13,14]. It was reported that exothermic polymerization and toxicity of the unreacted methyl methacrylate (MMA) cause local cellular death [15]. The mechanical properties of PMMA used in orthopedic applications play an influential role in determining the successful long-term stability of a prosthesis [16]. PMMA by itself possesses insufficient mechanical properties, which indeed increases the risk of crack formation and, therefore, implant loosening [8].

Incorporation of the reinforcement materials into the matrix of the PMMA bone cement is considered as one of the potential approaches for improving its bioactivity and mechanical properties [17,18]. For instance, bioactive reinforcement materials such as hydroxyapatite [19] and bioglass [20] have been incorporated into the matrix of the PMMA bone cement in the past. However, the results were not satisfactory since the addition of large amount of the reinforcement material deteriorated the mechanical properties of the composite while the minor quantity of the reinforcement was not effective on bioactivation of PMMA [21]. Recently, the application of nano-sized reinforcement materials into the PMMA matrix has attracted considerable attention [21]. For example, multiwalled carbon nanotube [22,23], graphene [24] calcium carbonate nanoparticles [25], collagen [26], silica nanoparticles [27], alumina nanoparticles [28], core-shell nanoparticles [29] and ZrO_2_ nanotubes [30] have been widely incorporated as the reinforcement materials into PMMA in order to address the aforementioned shortcomings. 

Recently, 2D nanomaterials have attracted significant interest as promising nanoplatforms for biomedical applications owing to their ultrathin thickness, specific physicochemical properties, high surface-area-to-mass ratio, and 2D morphological feature [31]. Magnesium phosphate nanosheets are a biocompatible and bioresorbable material in vivo, which accelerated bone healing and osseointegration [32]. The synthesis of the flexible hydroxyapatite nanofibers with the unique mechanical properties using a hydrothermal process has been reported [33]. The combination of the MgP nanosheets and the HA nanofibers has not been examined in the area of nanocomposite materials.

The purpose of this study is to investigate the synergistic effect of the HA nanofibers and 2D MgP nanosheets ratios on the mechanical property and the cytocompatibility of the PMMA nanocomposites. Therefore, synthesized the MgP nanosheets and the HA nanofibers were mixed into the PMMA matrix at three different ratios. Central composite design (CCD) as a useful statistical-based experimental design tool was employed for designing the experiment. The simultaneous effect of HA and MgP ratios on the ultimate compressive strength and cell viability of the PMMA-HA-MgP nanocomposites was investigated. The empirical models based on the effective factors and interactions were developed and applied as a prediction tool for optimization. In addition, the synthesized HA nanofibers, the MgP nanosheets, and the PMMA-HA-MgP nanocomposites were characterized using X-ray diffraction (XRD), transmission electron microscopy (TEM), field emission scanning electron microscope (FESEM) and energy-dispersive X-ray spectroscopy (EDS). 

## 2. Material and Methods

### 2.1. Synthesis of the HA Nanofibers

In a typical procedure, the aqueous solution of calcium nitrate tetrahydrate (0.01 M) as the calcium (Ca) source, diammonium hydrogen phosphate (0.06 M) as the phosphorus (P) source, and urea (1 M) solution were first mixed together. The molar ratio of Ca/P was kept at 1.67. HNO_3_ (0.5 M) was then added to this solution until its pH value reached 3.50. After stirring for 30 min, the solution was transferred to a 100 mL stainless-steel autoclave and was treated hydrothermally at 160 °C for 6 h. Finally, the products were centrifugally cleaned with deionized water and absolute ethanol and then dried in an oven at 80 °C for 5 h [34].

### 2.2. Synthesis of 2D MP Nanosheets

According to the study reported by Laurenti et al., [30], a ternary system of NaOH–Mg(OH)_2_–H_3_PO_4_ was employed to synthesize two dimensional MgP nanosheets. First, the MgOH (1 M) was dissolved with the magnetic stirrer in H_3_PO_4_ (1 M) until a fully clear solution was obtained. Thereafter, the NaOH solution was added and standed for 2 h. Finally, the prepared solution was centrifuged at 4000 rpm for 5 min, and the supernatant was discarded. The molar ratios of MgOH, NaOH and H_3_PO_4_ were adjusted to 0.18, 0.45, and 0.37, respectively. The solid precipitate was vacuum dried at 70 °C for 24 h.

### 2.3. PMMA-HA-MgP Nanocomposite Preparation

The PMMA-HA-MgP nanocomposites were prepared by mixing different ratios of the HA nanofibers and the MgP nanosheets in PMMA according to the experimental design in Section 2.4. To prepare the nanocomposites, methyl methacrylate (MMA) was utilized as solvent for PMMA. The ratio of PMMA/MMA was set to the 1.52 g/mL [6]. The mixed powders and MMA were mixed together under ambient conditions (22 ± 1 °C) and at a relative humidity of not <40%. To ensure homogenous dispersion, the solutions were sonicated in triplicate at amplitude of 10 ± 1 μA for 30 ± 1 s. All tests specimens were fabricated using PTFE molds. The mixed components were injected into each PTFE molds at 45 ± 1 s. The prepared nanocomposites were then vacuum-dried for 36 h. To dry any remaining liquid, the samples were then oven-dried at 40 °C for 24 h.

### 2.4. Experimental Design

Response surface methodology (RSM) is defined as a collection of the statistical and mathematical techniques that can be employed to determine the effect of several factors at different levels and further their effects on each other [35]. This technique is suitable for analyzing and modeling for a specific application where a response of interest is influenced by different variables [36,37]. The objective of RSM is to optimize the response through adjusting the values of factors [38]. The first step in RSM is to find a suitable approximation for the functional relationship between independent variables “*X*” and dependent variable “*Y*”. Generally, a low order polynomial is employed in some of the independent variables; however, a polynomial of the higher degree such as the second order model is employed when there is curvature in the system as stated in Equation (1). [39]:

(1) $$Y=\beta_{0\;}+\overset{k}{\underset{i=1}{\sum }}\beta_{i\;}X_{i\;}+\overset{k}{\underset{i=1}{\sum }}\beta_{i}X_{i}^{2}\overset{k}{\underset{i=1}{\sum }}\overset{k}{\underset{j}{\sum }}\beta_{ij\;}-X_{i\;}-X_{j}+\ldots +e$$

where, *i*, *j*, *b*, *k*, and *e* stand for the linear coefficients, the quadratic coefficients, the regression coefficients, the number of experimental factors, and the random error, respectively.

In this study, a standard RSM design known as CCD was employed using Design of Expert software version 10 (Stat-Ease, Minneapolis, MN, USA) to investigate the simultaneous effect of independent factors including the HA nanofibers and the MgP nanosheets ratios on the ultimate compressive strength (UCS) and cell viability of PMM-HA-MgP nanocomposite. CCD is suitable for fitting a quadratic model, which usually works well for predication and optimization [40]. Among different types of CCD design, CCF was used owing to three levels of each factor including the high level (+1), the low level (−1), and the center points (coded as level 0). 

Table 1 represents the low, center, and high levels of the HA nanofibers and the MgP nanosheets ratios. The lowest and highest ratios were set to 2.5% wt and 7.5% wt, respectively, to ensure a lower degree of agglomeration in the reinforcements. In CCF design, alpha = ±1, and the star points are at the center of each face of the factorial space. This design includes two replications of factorial points, one replication of axial (star) point, and five center points. A total of 18 runs for this experimental plan and the obtained results are listed in Table 2. 

### 2.5. Compression Tests

According to ISO 5833 standard [41], the cylindrical shapes of each specimen with the length of 12.0 ± 0.1 mm and diameter of 6.0 ± 0.1 mm were tested under the compressive loading using an INSTRON–8500R universal testing machine. The machine operated at a crosshead speed of 2.54 mm/min until the specimen failed. 

### 2.6. Cytotoxicity Tests

In this study, cytotoxicity of PMMA-HA-MgP nanocomposites was evaluated using the MTT assay method. First, fibroblasts cells (3T3-J2) were cultured in Dulbecco’s modified eagle medium (DMEM) with addition of 10% fetal bovine serum (FBS) and 1% penicillin streptomycin followed by incubation at 37 °C with 5% CO_2_ and 95% humidity. Three disk shape samples of nanocomposites with dimension of 6 × 2 mm (D × W) were placed in a 96-well plate. Cultured cells were then seeded into the plate with the concentration of 10,000 cells per well followed by incubation for 24 h. MTT solution was prepared by diluting MTT in PBS with a concentration of 5 mg/mL. Subsequently, MTT solution was added, and the well-plate was incubated for 4 h. Dimethyl sulfoxide (DMSO) solvent was then added to each well to solubilize formazan salts. At the end, absorption was measured by a microplate reader (Synergy™ H1, BioTek, Winusky, VT, USA) at 570 nm wavelength.

### 2.7. Structural and Chemical Characterizations

Scanning electron microscope (SEM) (Hitachi S-3000N VPSEM) equipped with energy-dispersive X-ray spectroscopy (EDS) and field emission scanning electron microscope (FESEM) (JEOL JSM-6320F) were used to investigate surface morphology and elemental compositions. In addition, the nanostructures of the HA nanofibers and the 2D were observed under scanning transmission electron microscopy (STEM) (JEOL JEM-ARM200CF). The XRD patterns were recorded using a Bruker D8 Discover X-ray diffraction system equipped with a copper sealed X-ray tube source, producing Cu-Kα (λ = 1.5418 Å). The diffractometer was operated at 40.0 kV and 40.0 mA at a 2θ range of 5–60° with a step size of 0.02 and an exposure time of 1 s/step.

## 3. Results and Discussion 

**Figure 1:**
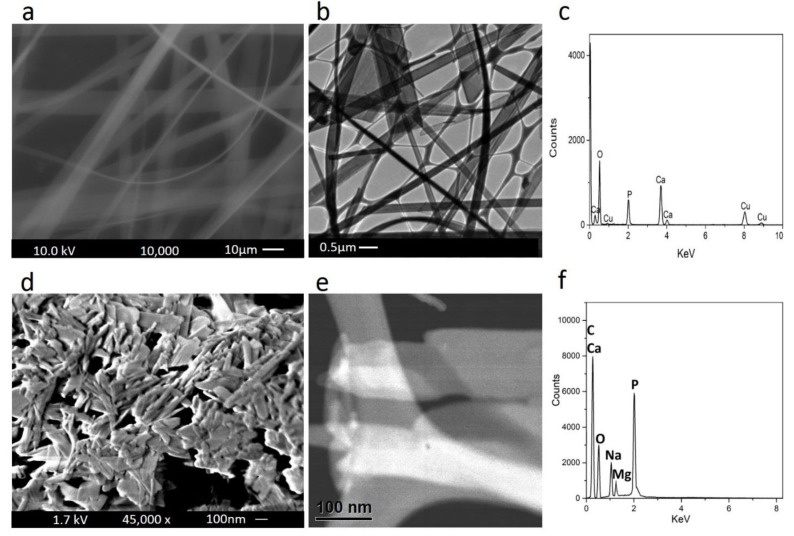
FESEM image, TEM image, and EDS spectra of (**a**–**c**) HA nanofibers and (**d**–**f**) MgP nanosheets.

Figure 1f shows electron microscopy, energy-dispersive X-ray spectroscopy, and elemental analysis of the HA nanofibers as well as the MgP nanosheets. SEM and TEM images of the HA nanofibers are shown in Figure 1a,b. SEM evaluation indicates the formation of uniform and solid nanofibers with random orientations. TEM image revealed that nanofibers were formed at high aspect ratios (micro meter length and nanometer diameter). 

It was reported that the optimal fiber used as reinforcement in a polymer-based composite should have high aspect ratio so that the stresses developed within them are remarkably larger than the nominal stresses in the composites [23]. Therefore, these HA nanofibers possibly fulfill the requirements of optimal fibers in the composite. The EDS analysis of the HA nanofibers (Figure 1c) indicates the existence of Ca, P, and O elements, which has good agreement with the findings of Qi et al. [34]. Figure 1d,e shows the morphology and nanostructure of the 2D magnesium phosphate nanosheets characterized by FESEM and TEM images. The sheet-like nanostructure with variable dimensions forms a continuous network containing several ultrathin layers of aggregated and crumpled sheets. The EDS analysis (Figure 1f) confirms that the 2D magnesium phosphate nanosheets consists of O, C, P, Mg, and Na elements, which has good agreement with the results of Laurenti et al. [42]. 

**Figure 2:**
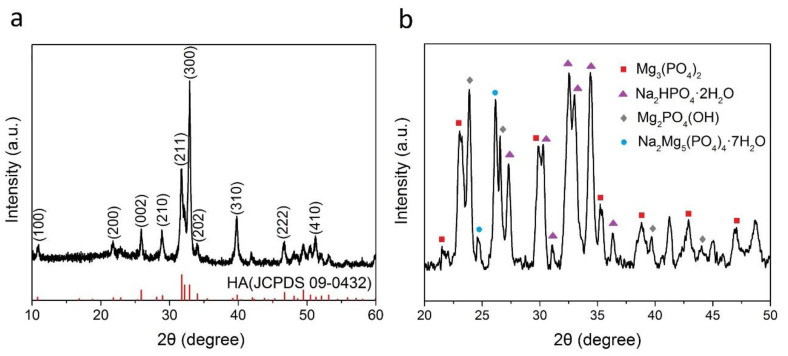
X-ray diffraction patterns of (**a**) the HA nanofibers (2θ = 10–60 degree) and (**b**) the MgP nanosheets (2θ = 20–50 degree). The red-colored peaks in Figure 2b indicate the standard HA from JCPDS 09-0432.

X-ray diffraction patterns of the HA nanofibers and the MgP nanosheets are shown in Figure 2a,b. All the peaks in Figure 2a can be indexed to HA (JCPDS 09-0432), and no other phase was detected indicating that the synthesized nanofibers are pure HA. This result is similar to those obtained by Qi et al. [34]. The diffraction peaks of the MgP nanosheets (Figure 2b) indicate that this component is mainly composed of Mg_3_(PO_4_)_2_, Na_2_HPO_4_·2H_2_O, Mg_2_PO_4_(OH), Na_2_Mg_5_(PO_4_)_4_·7H_2_O compounds. 

**Figure 3:**
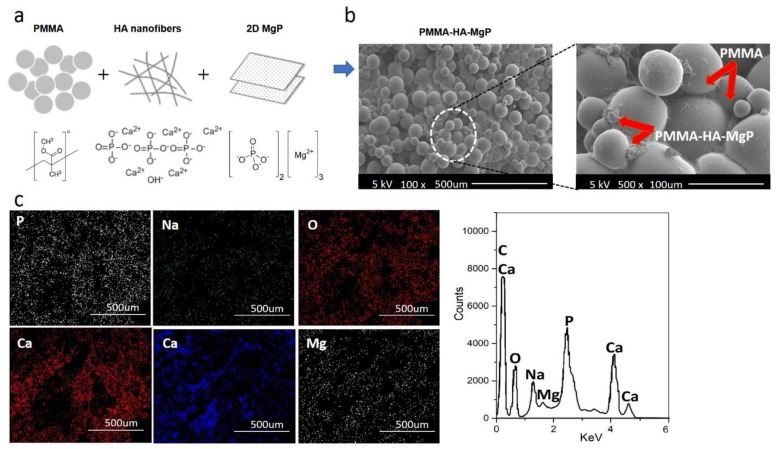
Characterization of the PMMA-MgP-HA nanocomposites: (**a**) schematic diagram for the formation of nanocomposite, (**b**) the SEM images in two different magnifications, and (**c**) the EDS mapping mode and the EDS spectrum.

Figure 3a shows the schematic diagram for developing the PMMA-based nanocomposite reinforced with the HA nanofibers and the MgP nanosheets. Surface morphology of the PMMA-HA-MgP nanocomposite was investigated through SEM images at two different magnifications (Figure 3b). From the SEM images, it is observed that the incorporation of two reinforcements in the PMMA matrix fills up the porosity between spherical particles of PMMA which results in a denser structure. In addition, the EDS mapping mode and the EDS spectra (Figure 3c) confirm that the developed nanocomposite consists of O, C, Mg, P, Ca, and Na elements with a homogenous distribution throughout the nanocomposite.

**Figure 4:**
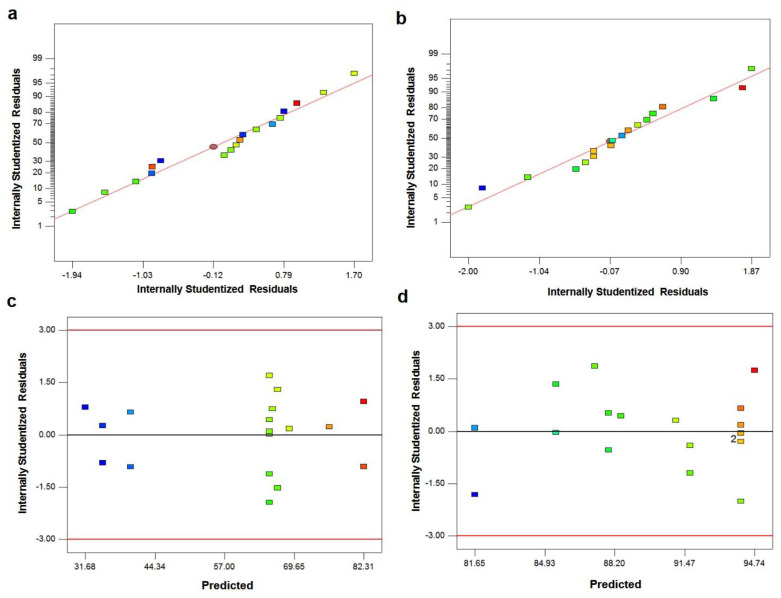
The normal probability plot associated with residuals and the plot associated with residuals vs. predicted response: (**a**,**c**) UCS and (**b**,**d**) cell viability.

**Figure 5:**
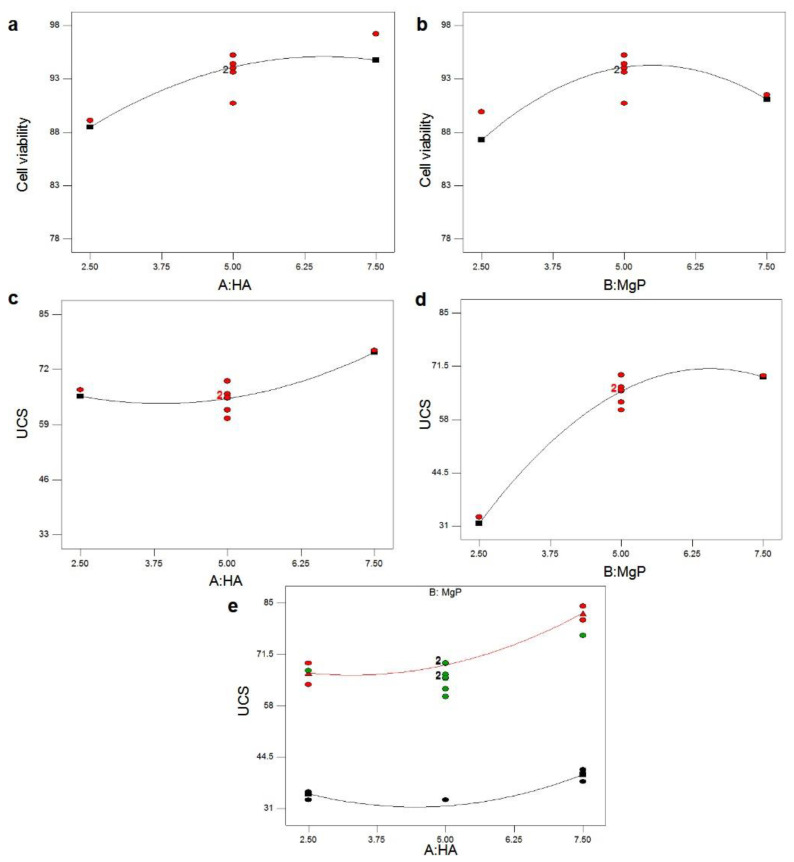
(**a**,**b**) The main effect plot for cell viability and (**c**–**e**) the main effect plot and interaction plot for UCS.

The normal probability plot of residuals for UCS and cell viability of PMMA-HA-MgP nanocomposite is shown in Figure 4a,b. It is shown that the residuals fall on a straight line in both normal probability plots. This indicates that the errors are distributed normally [43,44]. Figure 1c,d shows plot of residuals vs. predicted response for UCS and cell viability. The unusual structures or the unclear patterns of both plots imply that there is no reason to suspect any violation of the independence or constant variance assumption [45]. It means that the proposed models for both UCS and cell viability are adequate. Figure 5 illustrates the main effect plots for cell viability and UCS. As shown in Figure 5a, with the increase of the HA nanofibers ratio in the PMMA nanocomposite, the percentage of cell viability increases. In other words, the cytotoxicity of PMMA-HA-MgP nanocomposite decreased when the HA nanofibers increased. This growth in cell viability is more significant by increasing of HA nanofibers ratio from 2.5% wt to 5% wt. Figure 5b confirms that the cell viability of PMMA-based nanocomposite increased with the increase of MgP nanosheets from 2.5% wt to 5% wt. However, the incorporation of MgP nanosheets of more than 5% wt leads to reduction of cell viability. This is possibly related to the interaction between nanofibers and nanosheets and the character of the interface between the fiber–sheet system and PMMA matrix. The main effect plot and interaction plot for UCS is shown in Figure 5c–e. It seems that there is no significant effect on UCS of the PMMA-based nanocomposite with the increase of HA nanofibers from 2.5% wt to 5% wt. However, the HA nanofibers of more than 5% wt result in higher ultimate compressive strength. The set of nanofibers within this ratio increase the porosity of nanocomposite and possibly act as a deformation lock, therefore improving the mechanical properties. There is a sever increase in UCS when MgP ratio rises from 2.5 to 5% wt. However, the effect of MgP is not significant when its ratio is more than 5% wt. Findings reveal that an optimum amount of MgP can promote the mutual interaction between spherical PMMA particles (Figure 3b) and consequently enhance the mechanical properties. Figure 4c shows that there is an interaction effect between the HA ratios and the MgP ratio in the UCS of the PMMA nanocomposite. This indicates that the difference in the response between the levels of one factor is not the same at all levels of the other factors [39]. Figure 5a,b reveals the 3D surface graphs for UCS and cell viability of PMMA nanocomposite. Both show a curvilinear profile in accordance with the quadratic model fitted. As the model is adequate, these 3D surface graphs can be used for predicting the UCS and cell viability values for any suitable combination of the input parameters, namely, HA nanofibers and MgP nanosheets ratios. Generally, it is clear from graphs that the UCS increases with increasing HA and MgP ratios. In addition, the highest cell viability can be obtained for a HA and MgP ratios at a certain point. 

The normal probability plot of the residuals for UCS and cell viability of the PMMA-HA-MgP nanocomposite is shown in Figure 4a,b. It is shown that the residuals fall on a straight line in both normal probability plots. This indicates that the errors are distributed normally [43,44]. Figure 1c,d shows the plot associated with residuals vs. predicted response for UCS and cell viability. The unusual structures or unclear patterns of both plots imply that there is no reason to suspect any violation of the independence or constant variance assumption [45]. It means that the proposed models for both UCS and cell viability are adequate.

Figure 5a–e shows the main effect plots for cell viability and UCS. As shown in Figure 5a, with the increase of the HA nanofibers ratio in the PMMA nanocomposite, the percentage of cell viability increases. In other words, the cytotoxicity of the PMMA-HA-MgP nanocomposite decreased when the HA nanofibers increased. This growth in cell viability is more significant by an increase in the HA nanofibers ratio from 2.5 to 5% wt. Figure 5b confirms that the cell viability of the PMMA-based nanocomposite increased with the increase of the MgP nanosheets from 2.5 to 5% wt. However, the incorporation of the MgP nanosheets with more than 5% wt leads to a reduction of cell viability. This is possibly related to the interaction between nanofibers and nanosheets and the character of the interface between the fiber-sheet system and the PMMA matrix. 

The main effect plot and the interaction plot for UCS are shown in Figure 5c–e. It is evident that there is no significant effect on UCS of the PMMA-based nanocomposite with the increase of the HA nanofibers from 2.5 to 5% wt. However, the HA nanofibers with more than 5% wt result in the higher ultimate compressive strength. The set of nanofibers within this ratio increase the porosity of nanocomposite and possibly act as a deformation lock, therefore improving the mechanical properties. There is a sharp increase in UCS when the MgP ratio rises from 2.5 to 5% wt. However, the effect of MgP is not significant when its ratio is more than 5% wt. This finding demonstrates that the optimum amount of MgP can promote the mutual interaction between the spherical PMMA particles (Figure 3b) which ultimately enhances the mechanical properties. Figure 5c shows that there is an interaction effect between the HA ratios and the MgP ratio in the UCS of the PMMA nanocomposite. This indicates that the difference in the response between the levels of one factor is not the same at those of the other factors [39]. 

**Figure 6:**
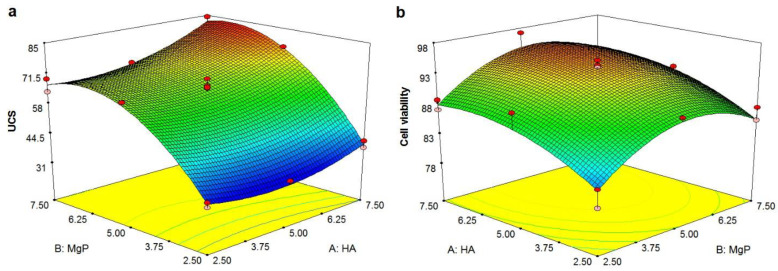
The 3D surface graph: (**a**) UCS and (**b**) cell viability. (Note: it shows the relationship between a response variable *Z* and two independent factors *X* and *Y*).

Figure 6a,b reveals the 3D surface graphs for UCS and cell viability of the PMMA nanocomposite which indicate a curvilinear profile in accordance to the quadratic model fitted. Since the model is adequate, these 3D surface graphs can be used for predicting the UCS and cell viability values for the any suitable combination of the input parameters known as the HA nanofibers and the MgP nanosheets ratios. Generally, it is clear from graphs that the UCS increases with increasing the HA and the MgP ratios. In addition, the highest cell viability can be obtained for the HA and the MgP ratios at a certain point.

The ANOVA table for UCS is summarized in Table 3. Using the variance of all the terms at an appropriate level α, the F-value of the model determines its significance [46]. The F-value of this model is 127.78, implying that it is significant. There is only a 0.01% chance that a Model F-Value could occur due to the noise. Values of Prob >F less than 0.05 indicate that the result is not random and the term model has a significant effect on the response [47]. Therefore, A, B, AB, A^2^, and B^2^ are significant factors. The “Lack of Fit F-value” of 0.26 implies there are 85.23 chances that it could occur due to noise. The Pred R-Squared of 0.9816% is in reasonable agreement with the Adj R-Squared of 0.9739. This indicates that this regression model can reasonably predict responses for new observations. 

Adeq Precision measures the signal to noise ratio, and a ratio greater than four is desirable. In this model, the ratio is 33.121, implying an adequate signal. Therefore, this model can be used to navigate the design space. The coefficient of variation (CV), which is a ratio of the standard deviation to the mean, is 4.41%. This amount is lower than 10%, implying that the model is reproducible. The ANOVA table for cell viability is summarized in Table 4. The F-value of the model is 21.46, implying that this model is significant. There is only a 0.01% chance that a Model F-Value could occur due to the noise. In this model, A, B, A^2^, and B^2^ are significant factors because their values of “Prob > F” are less than 0.05. However, AB is a nonsignificant factor because its value is greater than 0.1. In order to improve the model, the nonsignificant terms need to be removed. Therefore, it is essential to apply the model reduction. Table 5 presents the ANOVA table for the reduced quadratic model. AB, which was not a significant factor, was removed in this Table. The F-value of the model is 24.53, indicating that the model is still significant, and there is only a 0.01% chance that a Model F-Value could occur due to noise. 

The “Lack of Fit F-value” of 2.61 implies there is a 10.64% chance that a “Lack of Fit F-value” could occur due to the noise. The Pred R-Squared of 0.8830 has good agreement with the Adj R-Squared of 0.8470. Adeq Precision measures the signal to the noise ratio, and a ratio greater than four is desirable. In this model, the ratio is 13.559 implying an adequate signal. This model can be used to navigate the design space. The CV of this model is 2.04%, which is lower than 10%, implying that this model is also reproducible. Final equations in terms of actual factors for the UCS and cell viability are presented in Equations (2) and (3). The adequacy of these developed models needs to be assessed prior to their use in the optimization process.

UCS = − 8.47000 − 9.20897A + 29.17103B + 0.42600AB+ 0.91510A^2^ − 2.38890B^2^ (2)

Cell viability = 54.39 + 5.22103A + 8.64903B − 0.39690A^2^ − 0.78890B^2^ (3)

where, A is the HA nanofibers, and B is the MgP nanosheets.

The adequacy of the developed models for UCS and cell viability was assessed by six confirmation tests. The conditions for the first three confirmation run experiments are among the conducted tests, and the last three confirmation tests are not previously performed but are within the previously defined levels. Table 6 presents the confirmation tests for the ultimate compressive strength. The percentage error range between the actual and predicted values for UCS is 0.6–7.3%. The confirmation test for cell viability is presented in Table 7. 

The residuals between the actual and predicted values for cell viability indicate that the percentage errors are between 0–4.5%. The confirmation tests revealed good correlation between the predicted and the experimental values with less than 10% of error, which is acceptable. Therefore, it can be said that the empirical models derived from RSM are reasonably accurate and, thereby, can be employed in the optimization process. 

The optimization process determines the combination of the dependent factor levels that simultaneously satisfy the sought requirements [48]. Optimal selection based on the developed models is the main objective to concurrently achieve the high performance of the PMMA nanocomposite in terms of the mechanical properties and cytotoxicity. Therefore, based on the numerical optimization in DOE software, the ratios of the HA nanofibers and the MgP nanosheets were set to “in arrange” due to no preference for the value. The UCS and cell viability were set to be maximized. Based on this goal setting, the possible optimal solution suggested by the software for dependent factors is presented in Table 8. 

**Figure 7:**
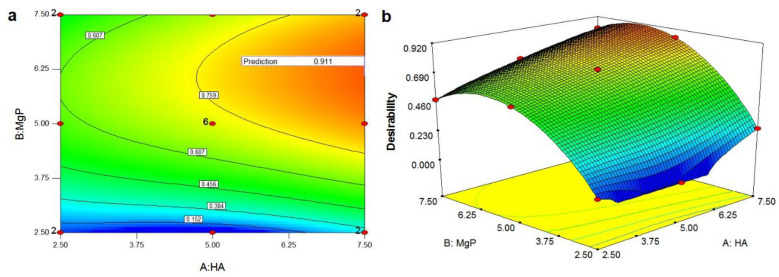
Overall desirability function: (**a**) contour plots and (**b**) 3D plots. (Note: The optimum region is in the red area of the graph, which has an overall desirability value of 0.911.)

The PMMA-HA-MgP with the HA nanofibers ratio of 7.5% wt and the MgP nanosheets ratio of 6.12% wt reveals the maximum UCS and cell viability with the highest level of desirability (91.1%). Figure 7a,b shows the overall desirability function including contour plots and the 3D plots. A contour plot is produced to visually display the region of optimal factor settings [49]. By generating contour plots using DOE software for response surface analysis, the optimum is located with reasonable accuracy. In this study, the optimum region is inspected to be in the upper right region of the graph, which has an overall desirability value of 0.911. The optimum region gradually shrinks by moving toward the lower left region of the plot.

## 4. Conclusions

In this study, the HA nanofibers and the MgP nanosheets as the reinforcement materials were synthesized through hydrothermal homogeneous precipitation and tuning of the crystallization of the sodium–magnesium–phosphate ternary system, respectively. A novel PMMA bone cement nanocomposite was then developed by mixing the HA nanofibers and the MgP nanosheets at different ratios of reinforcement. A standard RSM design known as CCD was employed to investigate the effect of the HA nanofibers and the MgP nanosheets ratios on the ultimate compressive strength and cell viability of the PMM-HA-MgP nanocomposite.

The results demonstrated that both the HA nanofibers and the MgP nanosheets are considered as two significant factors to improve UCS. However, the effect of the MgP nanosheets was observed to be more significant. In addition, both reinforcements are significant factors for enhancing the cytocompatibility of the PMMA-based nanocomposite under the influence of the higher amounts of the HA nanofibers. A set of confirmation tests demonstrated that the empirical models derived from RSM can be used to describe the relationship between the independent factors and responses. The optimization results demonstrated that the maximum UCS and cell viability of the PMMA-HA-MgP nanocomposite is obtained with the HA nanofibers ratio of 7.5% wt and the MgP nanosheets ratio of 6.12% wt with the highest level of desirability (91.1%). 

The current result is based on the selected ratios of reinforcements. Therefore, more experiments need to be conducted at different ranges of ratio. In addition, more research needs to be conducted to investigate the mechanism of this nanocomposite. 

## Figures and Tables

**Table 1:** HA and MgP ratio Factors and their levels.

Levels	Factors	
	HA Ratio (% wt) A	MgP Ratio (% wt) B
High (1)	7.5	7.5
Low (−1)	2.5	2.5
Centre point (0)	0	0

**Table 2:** The experimental plan and their associated results for surface response methodology.

Std. no.	Run	HA (% wt)	MgP (% wt)	UCS (MPa)	Cell Viability (%)
1	6	2.50	2.50	33.3	81.8
2	13	2.50	2.50	35.4	78.9
3	10	7.50	2.50	38.1	88.7
4	12	7.50	2.50	41.2	87.1
5	4	2.50	7.50	69.2	85.4
6	17	2.50	7.50	63.6	87.5
7	14	7.50	7.50	84.2	91.1
8	3	7.50	7.50	80.5	89.9
9	16	2.50	5.00	67.2	89.1
10	2	7.50	5.00	76.5	97.2
11	5	5.00	2.50	33.3	89.9
12	8	5.00	7.50	69.1	91.5
13	15	5.00	5.00	65.2	94.4
14	7	5.00	5.00	62.4	95.2
15	11	5.00	5.00	60.4	93.6
16	9	5.00	5.00	66.2	90.7
17	18	5.00	5.00	69.3	94
18	1	5.00	5.00	65.4	93.6

**Table 3:** ANOVA table for the quadratic model (UCS).

Source	Sum of Squares	Df	Mean Square	F Value	*p*-Value Prob > F	
**Model**	4477.32	5	895.46	127.78	<0.0001	significant
**A-HA**	268.32	1	268.32	38.29	<0.0001	
**B-MgP**	3433.61	1	3433.61	489.96	<0.0001	
**AB**	56.71	1	56.71	8.09	0.0148	
**A^2^**	101.40	1	101.40	14.47	0.0025	
**B^2^**	691.06	1	691.06	98.61	<0.0001	
**Residual**	84.10	12	7.01			
**Lack of Fit**	6.71	3	2.24	0.26	0.8523	not significant
**Pure Error**	77.38	9	8.60			
**Cor Total**	4561.42	17				
**Std. Dev.**	2.65		**R-Squared**	0.9816		
**Mean**	60.03		**Adj R-Squared**	0.9739		
**C.V. %**	4.41		**Pred R-Squared**	0.9611		
**PRESS**	177.33		**Adeq Precision**	33.121		

**Table 4:** ANOVA table for the reduced quadratic model (cell viability).

Source	Sum of Squares	df	Mean Square	F Value	*p*-Value Prob > F	
**Model**	335.39	5	67.08	21.46	<0.0001	significant
**A-HA**	97.97	1	97.97	31.35	0.0001	
**B-MgP**	36.10	1	36.10	11.55	0.0053	
**AB**	6.12	1	6.12	1.96	0.1868	
**A^2^**	19.08	1	19.08	6.10	0.0295	
**B^2^**	75.36	1	75.36	24.12	0.0004	
**Residual**	37.50	12	3.13			
**Lack of Fit**	17.32	3	5.77	2.58	0.1187	not significant
**Pure Error**	20.18	9	2.24			
**Cor Total**	372.89	17				
**Std. Dev.**	1.77		**R-Squared**	0.8994		
**Mean**	89.98		**Adj R-Squared**	0.8575		
**C.V. %**	1.96		**Pred R-Squared**	0.7578		
**PRESS**	90.30		**Adeq Precision**	13.683		

**Table 5:** ANOVA table for the reduced quadratic model (cell viability).

Source	Sum of Squares	df	Mean Square	F Value	*p*-Value Prob > F	
**Model**	329.27	4	82.32	24.53	<0.0001	significant
**A-HA**	97.97	1	97.97	29.19	0.0001	
**B-MgP**	36.10	1	36.10	10.76	0.0060	
**A^2^**	19.08	1	19.08	5.68	0.0330	
**B^2^**	75.36	1	75.36	22.46	0.0004	
**Residual**	43.63	13	3.36			
**Lack of Fit**	23.45	4	5.86	2.61	0.1064	not significant
**Pure Error**	20.18	9	2.24			
**Cor Total**	372.89	17				
**Std. Dev.**	3.87		**R-Squared**	0.9920		
**Mean**	51.63		**Adj R-Squared**	0.9891		
**C.V. %**	7.50		**Pred R-Squared**	0.9808		
**PRESS**	686.26		**Adeq Precision**	44.496		

**Table 6:** The confirmation tests for UCS.

No.	HA (% wt)	MgP (% wt)	Actual UCS (Mpa)	Predicted UCS (Mpa)	Residual	Error (%)
1	2.5	7.5	69.2	66.6	2.6	3.7
2	7.5	5	76.5	76	0.5	0.6
3	2.5	2.5	35.4	34.8	0.6	1.6
4	3.5	4	58.8	54.9	3.9	6.6
5	4.5	3	39.6	40.3	0.7	1.7
6	6	6.5	81.5	75.5	6	7.3

**Table 7:** The confirmation tests for cell viability.

No.	HA (% wt)	MgP (% wt)	Actual Cell Viability (%)	Predicted Cell Viability (%)	Residual	Error (%)
1	2.5	7.5	85.4	85.4	0	0
2	7.5	5	97.2	94.7	2.5	2.5
3	2.5	2.5	78.9	81.6	2.7	3.4
4	3.5	4	91.3	89.7	1.6	1.7
5	4.5	3	89.7	88.6	1.1	1.2
6	6	6.5	90.2	94.3	4.1	4.5

**Table 8:** The solution for optimization.

No.	HA (% wt)	MgP (% wt)	UCS (Mpa)	Cell Viability	Desirability
1	7.50	6.12	82.5307	94.6098	0.911
2	7.50	6.08	82.4215	94.6428	0.911

## Data Availability

The data that support the findings of this study are available from the corresponding author, upon reasonable request.
